# Transcriptome profile of rat genes in injured spinal cord at different stages by RNA-sequencing

**DOI:** 10.1186/s12864-017-3532-x

**Published:** 2017-02-15

**Authors:** Ling-Ling Shi, Nan Zhang, Xiu-Mei Xie, Yue-Juan Chen, Rui Wang, Lin Shen, Jian-Sheng Zhou, Jian-Guo Hu, He-Zuo Lü

**Affiliations:** 1 Clinical Laboratory, the First Affiliated Hospital of Bengbu Medical College, Anhui, 233004 People’s Republic of China; 2 Anhui Key Laboratory of Tissue Transplantation, the First Affiliated Hospital of Bengbu Medical College, 287 Chang Huai Road, Anhui, 233004 People’s Republic of China; 3 Department of Immunology, Bengbu Medical College, Anhui, 233030 People’s Republic of China

**Keywords:** Spinal cord injury, RNA-Seq, GO enrichment, Pathway analysis, Sprague-Dawley rats (RRID:RGD_70508)

## Abstract

**Background:**

Spinal cord injury (SCI) results in fatal damage and currently has no effective treatment. The pathological mechanisms of SCI remain unclear. In this study, genome-wide transcriptional profiling of spinal cord samples from injured rats at different time points after SCI was performed by RNA-Sequencing (RNA-Seq). The transcriptomes were systematically characterized to identify the critical genes and pathways that are involved in SCI pathology.

**Results:**

RNA-Seq results were obtained from total RNA harvested from the spinal cords of sham control rats and rats in the acute, subacute, and chronic phases of SCI (1 day, 6 days and 28 days after injury, respectively; *n* = 3 in every group). Compared with the sham-control group, the number of differentially expressed genes was 1797 in the acute phase (1223 upregulated and 574 downregulated), 6590 in the subacute phase (3460 upregulated and 3130 downregulated), and 3499 in the chronic phase (1866 upregulated and 1633 downregulated), with an adjusted *P*-value <0.05 by DESeq. Gene ontology (GO) enrichment analysis showed that differentially expressed genes were most enriched in immune response, MHC protein complex, antigen processing and presentation, translation-related genes, structural constituent of ribosome, ion gated channel activity, small GTPase mediated signal transduction and cytokine and/or chemokine activity. Kyoto Encyclopedia of Genes and Genomes (KEGG) pathway analysis showed that the most enriched pathways included ribosome, antigen processing and presentation, retrograde endocannabinoid signaling, axon guidance, dopaminergic synapses, glutamatergic synapses, GABAergic synapses, TNF, HIF-1, Toll-like receptor, NF-kappa B, NOD-like receptor, cAMP, calcium, oxytocin, Rap1, B cell receptor and chemokine signaling pathway.

**Conclusions:**

This study has not only characterized changes in global gene expression through various stages of SCI progression in rats, but has also systematically identified the critical genes and signaling pathways in SCI pathology. These results will expand our understanding of the complex molecular mechanisms involved in SCI and provide a foundation for future studies of spinal cord tissue damage and repair.

The sequence data from this study have been deposited into Sequence Read Archive (http://www.ncbi.nlm.nih.gov/sra; accession number PRJNA318311).

**Electronic supplementary material:**

The online version of this article (doi:10.1186/s12864-017-3532-x) contains supplementary material, which is available to authorized users.

## Background

Spinal cord injury (SCI) results in a devastating loss of motor and sensory functions. In the United States, it has been reported that some 300,000 people are living with SCI and nearly 12,000 new patients are diagnosed annually [1]. In China, the incidence of traumatic SCI is approximately 60,000 per year [2]. Despite advances in surgical techniques for the spinal cord, there are still no effective treatments for this devastating neurological disorder. Therefore, it is very important to further understand the molecular changes in SCI in order to develop a better therapeutic program.

The pathological changes following traumatic SCI can be divided into two processes: primary injury and secondary injury. Primary injury is the immediate effect of mechanical damage on the spinal cord, which directly damages various tissue components. Secondary injury is a cascade of effects triggered by the primary injury affecting multiple biological processes and producing extensive temporal changes in gene expression [3]. Considering the complexity of gene expression and signaling pathways, a global analysis is necessary to understand the molecular mechanisms and develop therapeutic strategies for SCI. During the last decade, cDNA microarray and genechip technologies have provided valuable insights into gene changes after SCI [4–6]. However, these technologies have suffered from limitations in resolution, dynamic range and accuracy [7]. With the advances in genome-wide transcriptome analysis, especially high-throughput RNA sequencing (RNA-Seq) technology, we now have a more useful approach to observe whole transcriptome changes [7, 8].

In this study, temporal genome-wide gene expression profiles in injured spinal cords of adult rats were examined using RNA-Seq, and the results were confirmed by real-time quantitative reverse-transcriptase polymerase chain reaction (qRT-PCR). Based on these data, the functions and pathways involved in the pathologic process of SCI in the acute, subacute, and chronic phases were characterized. The bioinformatics analysis confirmed that some of the expressed genes in our study have been shown to play important roles in SCI, thus supporting the reliability of our data. We also identified many additional expressed genes whose functions in SCI have not been well studied. These additional genes may be involved in the pathological process of SCI and deserve further study and discussion.

## Methods

### Animals

We used 12 female Sprague-Dawley rats (eight-week-old, 220–250 g, RRID: RGD_70508) in this study. Animal care in surgical procedures and after operation were in accordance with Regulations for the Administration of Affairs Concerning Experimental Animals (Ministry of Science and Technology, China; revised in June 2004) and Guidelines and Policies for Rodent Survival Surgery provided by the Animal Care and Use Committees of Bengbu Medical College.

### Contusive SCI

To perform the contusive SCI, a New York University Impactor was used as described [9]. Rats were anesthetized by using pentobarbital (50 mg/kg, intraperitoneal) and received a T9 vertebral laminectomy. Sham-operated (sham) rats only received a laminectomy without contusive injury. For making contusive injury, the spine was stabilized by clamping T7 and T11 spinous processes. A weight drop injury of the cord was made by using a 10 g rod (2.5 mm diameter) dropped at a height of 25 mm on the exposed dorsal surface. After injury, animals were placed in a humidity- and temperature-controlled chamber. In 3 consecutive days, buprenorphine (0.05 mg/kg, SQ; Reckitt Benckise, Hull, England) was used every 12 h to relieve pain. Bladder emptying was manually performed three times daily until autonomous urination was established. Chloramphenicol (50 ~ 75 mg/kg) was daily provided via drinking water to prevent infections.

### Basso, Beattie, and Bresnahan (BBB) locomotor rating scale

BBB locomotor rating scale was used to assess the hind-limb movements based on observation of the rat’s freely moving in an open field [10, 11]. The evaluations were performed by 2 scorers while rats were walking freely on the open-field surface for 4 min. The scores were evaluated before operation, at 1 day, 3, 6, 10, 14, 21, and 28 days after injury.

### RNA isolation, quantification, and qualification

Spinal cords (5 mm spinal cord segment containing the injury epicenter) of sham, acute, subacute, and chronic phases were harvested at 6 days, 1 day, 6 days, and 28 days after injury, respectively (*n* = 3 in every group). Rats were perfused with phosphate buffer saline (PBS) before removing the spinal cords. The total RNA from spinal cords was extracted using TRIzol reagent (Invitrogen, New Jersey, NJ, USA). Genomic DNA was removed by using DNase I. The 1% agarose gels were used to monitor RNA degradation and contamination. NanoPhotometer® spectrophotometer (IMPLEN, CA, USA) was used to check RNA purity. Qubit® RNA Assay Kit in Qubit® 2.0 Flurometer (Life Technologies, CA, USA) was used to measure RNA concentration. RNA Nano 6000 Assay Kit of the Bioanalyzer 2100 system (Agilent Technologies, CA, USA) was used to assess RNA integrity.

### Library preparation for transcriptome sequencing

For the sample preparations, 3 μg RNA was used as input material for every sample. NEBNext® Ultra™ RNA Library Prep Kit for Illumina® (NEB, USA) was used to generate sequencing libraries. For each sample, the index codes were added to attribute sequences. The poly-T oligo-attached magnetic beads were used to purify mRNA from total RNA. In NEBNext First Strand Synthesis Reaction Buffer (5×), the mRNA was fragmented by divalent cations under elevated temperature. M-MuLV Reverse Transcriptase (RNase H) and random hexamer primers were used to synthesize the first strand cDNA. RNase H and DNA polymerase I were subsequently used to synthesize the second strand cDNA. The exonuclease/polymerase activities converted remaining overhangs into blunt ends. To prepare for hybridization, the 3′ ends of DNA fragments were adenylated and ligated to NEBNext Adaptor with hairpin loop structure. AMPure XP system (Beckman Coulter, Beverly, USA) was used to purify the library fragments to select cDNA fragments of preferentially 150–200 bp. Before PCR, size-selected, adaptor-ligated cDNAs were treated with 3 μl USER Enzyme (NEB, USA) at 37 °C for 15 min followed by 95 °C for 5 min. Index (X) Primer, Universal PCR primers and Phusion High-Fidelity DNA polymerase were used to perform PCR. Finally, AMPure XP system was used to purify PCR products, and the Agilent Bioanalyzer 2100 system was used to assess library quality.

### Clustering and sequencing

TruSeq PE Cluster Kit v3-cBot-HS (Illumina) was used to cluster the index-coded samples on a cBot Cluster Generation System by Novogene (Beijing, China) according to the manufacturer’s instructions. Finally, the 125 bp/150 bp paired-end reads were generated and sequenced on an Illumina Hiseq platform.

### Data analysis

#### Quality control

To obtain high-quality clean data for downstream analyses, the low quality reads and reads containing adapters or ploy-N in raw data were removed and the Q20, Q30 and GC content of those clean data were also calculated.

### Reads mapping to the reference genome

From genome website, the files of reference genome and gene model annotation were downloaded. Bowtie v2.2.3 was used to build the reference genome index. TopHat v2.0.12 was used to align the paired-end clean reads to the reference genome.

### Gene expression level quantification

The reads numbers mapped to each gene were counted by HTSeq v0.6.1. Fragments Per Kilobase of transcript sequence per millions base pairs sequenced (FPKM) was calculated. FPKM is the most commonly used method for evaluating the sequencing depth and gene length of the reads and estimating the levels of gene expression [12].

### Analysis of differential expression

Differential expression of four groups (three biological replicates per group) was analyzed using the DESeq R package (1.18.0). To control the false discovery rate, Benjamini and Hochberg’s approach was used to adjust the *P*-values obtained from DESeq analysis. The adjusted *P*-value < 0.05 was adopted as the standard for judging statistically significant differences in gene expression.

### Gene ontology (GO) and Kyoto encyclopedia of genes and genomes (KEGG) enrichment analysis of differentially expressed genes (DEGs)

The GOseq R package was used to perform GO enrichment analysis of DEGs. A corrected *P*-value < 0.05 was adopted as the standard for judging statistically significant enrichment of differentially expressed genes. KEGG enrichment analysis of DEGs was implemented by KOBAS software.

### qRT-PCR

To validate RNA-Seq results, 13 selected DEGs were verified by qRT-PCR as described [13]. A reverse transcription system (Promega, Madison, WI) was used to reverse-transcribe the total RNA used in RNA-Seq into cDNA. SYBR Green PCR Master Mix and the ABI 7900 PCR detection system (Applied Biosystems, Foster City, CA) were used to perform real-time PCR. To normalize gene expression, the housekeeping gene glyceraldehyde-3-phosphate dehydrogenase (GAPDH) was parallel amplified. PCR primer sequences are listed in Table 1. The ^ΔΔ^Ct method [14] was used to calculate the relative expression level of target mRNAs. The relative mRNA expression value in sham group was designated as 1.

**Table 1:** Real-time PCR primers used in the study

Gene	GenBank accession no.	Forward primer 5′ - 3′	Reverse primer 5′ - 3′
C1qb	NM_019262.1	TTCACCTACCACGCCAGTTC	GCTTCAAGACTACCCCACCC
Ccl2	NM_031530.1	TGATCCCAATGAGTCGGCTG	GGTGCTGAAGTCCTTAGGGT
Cxcl2	NM_053647.1	CAGGGTACAGGGGTTGTTGT	AGGTCAGTTAGCCTTGCCTT
Enpp3	NM_019370.2	TGACTCCGGATTTGCCGAAA	CTTCGCAGTTGGAACTCCCT
Il6	NM_012589.2	ACCCCAACTTCCAATGCTCT	AGCACACTAGGTTTGCCGAG
Pf4	NM_001007729.1	TCCAGGATCCATCTCAAACGC	TTTGCTCCCATTCTTCAGCG
Tspo	NM_012515.2	GGTGGACCTCATGCTTGTCA	CCTCGCCGACCAGAGTTATC
Plau	NM_013085.3	GCGGAACTCCTATAACCCCG	GTGGCACTCTCTTGTCCGAA
Ncf1	NM_053734.2	CATTGAGGCCGGTGAGATCC	AGTTCAAGAGGTGTGGGCAG
Lcn2	NM_130741.1	CAAGTGGCCGACACTGACTA	CATTGGTCGGTGGGAACAGA

## Results

### BBB scores at different stages of SCI

**Fig. 1:**
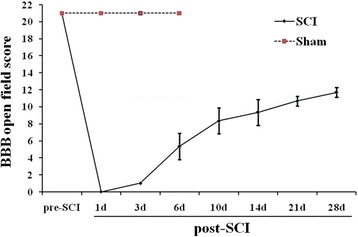
BBB scores at different stages of the SCI. The BBB scores were shown at different stages of the SCI: before injury (*n* = 12); sham (*n* = 3); 1 dpi (*n* = 9); 3 dpi and 6 dpi (*n* = 6); 10 dpi, 14 dpi, 21 dpi, and 28 dpi (*n* = 3)

To observe the level of impairment produced by SCI, BBB scores were evaluated. As shown in Fig. 1, all animals scored 21 points before injury. At 1 day post-SCI, all nine spinal cord-injured rats received a score of 0, while the three sham-operated animals still received scores of 21 points. On the 3^rd^ and 6^th^ days, the mean BBB scores of the six surviving injured rats were 1 ± 0 and 5.33 ± 1.53, respectively. After 6 days, with only three injured rats remaining, BBB scores had improved substantially. The mean scores at 10 days post-injury (dpi), 14 dpi, 21 dpi and 28 dpi were 8.33 ± 1.53, 9.33 ± 1.53, 10.67 ± 0.58, and 11.67 ± 0.58, respectively. According to our previous experience, this was a moderate level of impairment.

### Identification of expressed transcripts in the rat spinal cord transcriptome

In this study, we established 12 cDNA libraries with the following designations. Libraries with the A designation were from the sham-operated spinal cords and libraries B1, B2 and B3 were from injured spinal cords at 1 dpi, 6 dpi and 28 dpi, respectively, which represented of sham and three different pathological stages of SCI (acute, subacute, and chronic phases). RNA-Seq generated 41,724,966 to 58,619,478 raw reads for each library. After filtering the low-quality reads, the average number of clean reads was 42,505,205 (87.73%), 43,520,840 (82.37%), 43,336,187 (91.37%), and 42,483,112 (89.70%) for the A, B, C and D groups, respectively (Table 2). The clean reads were used for all further analyses, and from them 77.44% to 88.79% of clean tags from the RNA-Seq data mapped uniquely to the genome, while a small proportion of them (<3%) were mapped multiple times to the genome (Table 3).

**Table 2:** Summary of sequence assembly after Illumina sequencing

Sample name	Raw reads	Clean reads	clean bases	Error rate (%)	Q20 (%)	Q30 (%)	GC content (%)
A1	44881934	40093612	6.01G	0.02	95.33	88.31	50.28
A2	54272034	45508458	6.83G	0.02	97.31	92.22	50.48
A3	46199060	41913544	6.29G	0.02	96.3	90.38	49.91
B1	52270362	44613686	6.69G	0.02	97.09	91.43	50.31
B2	47612764	43741790	6.56G	0.02	96.03	89.63	50.16
B3	58619478	42207044	6.33G	0.02	98.35	93.93	50.37
C1	47746200	44041564	6.61G	0.02	96.17	89.9	50.37
C2	52818270	45368646	6.81G	0.02	97.14	91.56	50.88
C3	41724966	40598352	6.09G	0.03	94.79	87.81	50.96
D1	54673906	45455460	6.82G	0.02	96.48	89.84	50.48
D2	41816080	40410590	6.06G	0.03	94.79	87.89	50.6
D3	45601476	41583286	6.24G	0.02	95.94	89.46	50.51

A1- A3: sham; B1- B3: 1 dpi; C1- C3: 6 dpi; D1- D3: 28 dpi;

Q20: The percentage of bases with a Phred value > 20;

Q30: The percentage of bases with a Phred value > 30

**Table 3:** Summary of clean reads mapped to the reference genome

Sample name	A1	A2	A3	B1	B2	B3	C1	C2	C3	D1	D2	D3
Total reads	40093612	45508458	41913544	44613686	43741790	42207044	44041564	45368646	40598352	45455460	40410590	41583286
Total mapped	33174052 (82.74%)	39784929 (87.42%)	35390098 (84.44%)	39156701 (87.77%)	36809049 (84.15%)	38574060 (91.39%)	37395128 (84.91%)	40004868 (88.18%)	32801663 (80.8%)	39311658 (86.48%)	32232784 (79.76%)	34737783 (83.54%)
Multiple mapped	917254 (2.29%)	1067019 (2.34%)	946894 (2.26%)	1176321 (2.64%)	997260 (2.28%)	1098570 (2.6%)	1185829 (2.69%)	1369524 (3.02%)	1022149 (2.52%)	1119321 (2.46%)	937961 (2.32%)	1015029 (2.44%)
Uniquely mapped	32256798 (80.45%)	38717910 (85.08%)	34443204 (82.18%)	37980380 (85.13%)	35811789 (81.87%)	37475490 (88.79%)	36209299 (82.22%)	38635344 (85.16%)	31779514 (78.28%)	38192337 (84.02%)	31294823 (77.44%)	33722754 (81.1%)
Non-splice reads	20272875 (50.56%)	24409418 (53.64%)	22061282 (52.64%)	23293716 (52.21%)	22443212 (51.31%)	22884473 (54.22%)	21414897 (48.62%)	21880241 (48.23%)	18591284 (45.79%)	22881690 (50.34%)	18665456 (46.19%)	20269939 (48.75%)
Splice reads	11983923 (29.89%)	14308492 (31.44%)	12381922 (29.54%)	14686664 (32.92%)	13368577 (30.56%)	14591017 (34.57%)	14794402 (33.59%)	16755103 (36.93%)	13188230 (32.48%)	15310647 (33.68%)	12629367 (31.25%)	13452815 (32.35%)

### Identification of the source of variance in the expressed transcripts by principal component analysis (PCA)

**Fig. 2:**
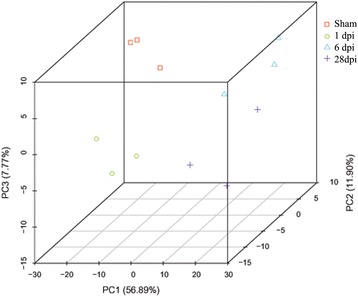
PCA analysis of the expressed transcripts. PCA analysis with three principal components (PC1, 2, and 3) was performed to demonstrate the source of variance in our data (*n* = 3)

To demonstrate the source of variance in our data, PCA analysis with three principal components (PC1, 2, and 3) was performed. As shown in Fig. 2, PC score plots showed that the contribution of PC1, 2, and 3 was 56.89%, 11.90%, and 7.77%, respectively. The three individual samples collected at the same time points after injury were clustered closely together which validated the finding of low variance in the present analysis study and showed that the data could be used for the following analysis.

### Differential gene expression in spinal cords at different injury stages

**Fig. 3:**
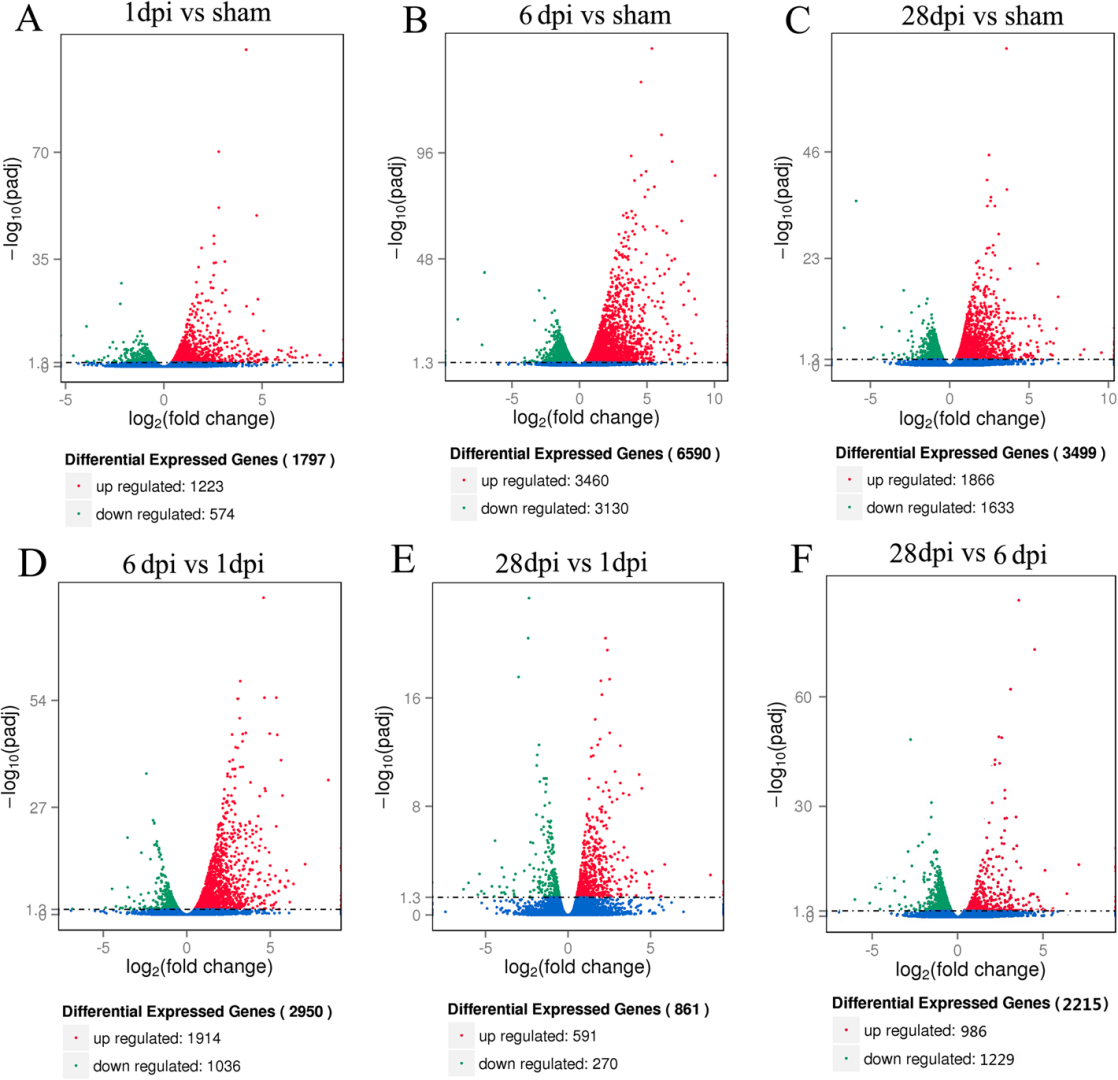
Volcano map of differentially expressed genes. Significantly upregulated and downregulated genes are shown as a red and green dot, respectively. The blue dot represents no significant difference between the expressions of genes. **a** 1 dpi vs sham, **b** 6 dpi vs sham, **c** 28 dpi vs sham, **d** 6 dpi vs 1 dpi, **e** 28 dpi vs 1 dpi, **f** 28 dpi vs 6 dpi

RPKM was used to estimate the level of gene expression, and DEGSeq was used to examine the differential gene expression profile. Comparing the SCI and sham animals, we observed 1,979 DEGs at 1 dpi, with 1,223 upregulated and 574 downregulated genes (Fig. 3a and Additional file 1: Table S1), 6590 DEGs at 6 dpi, with 3460 upregulated and 3130 downregulated genes (Fig. 3b and Additional file 1: Table S1) and 3499 DEGs at 28 dpi, with 1866 upregulated and 1633 downregulated genes (Fig. 3c and Additional file 1: Table S1). There were 2950 DEGs between the 6 dpi and 1 dpi groups, with 1914 upregulated and 1036 downregulated genes (Fig. 3d and Additional file 1: Table S1). There were 861 DEGs between the 28 dpi and 1 dpi groups, with 591 upregulated and 270 downregulated genes (Fig. 3e and Additional file 1: Table S1). There were 2215 DEGs between the 28 dpi and 6 dpi groups, with 986 upregulated and 1229 downregulated genes (Fig. 3f and Additional file 1: Table S1). A list of the most relevant genes that were over-expressed or under-expressed and how their expression changed with time is shown in Additional file 2: Table S2.

### Confirmation of differential gene expression by qRT-PCR

**Fig. 4:**
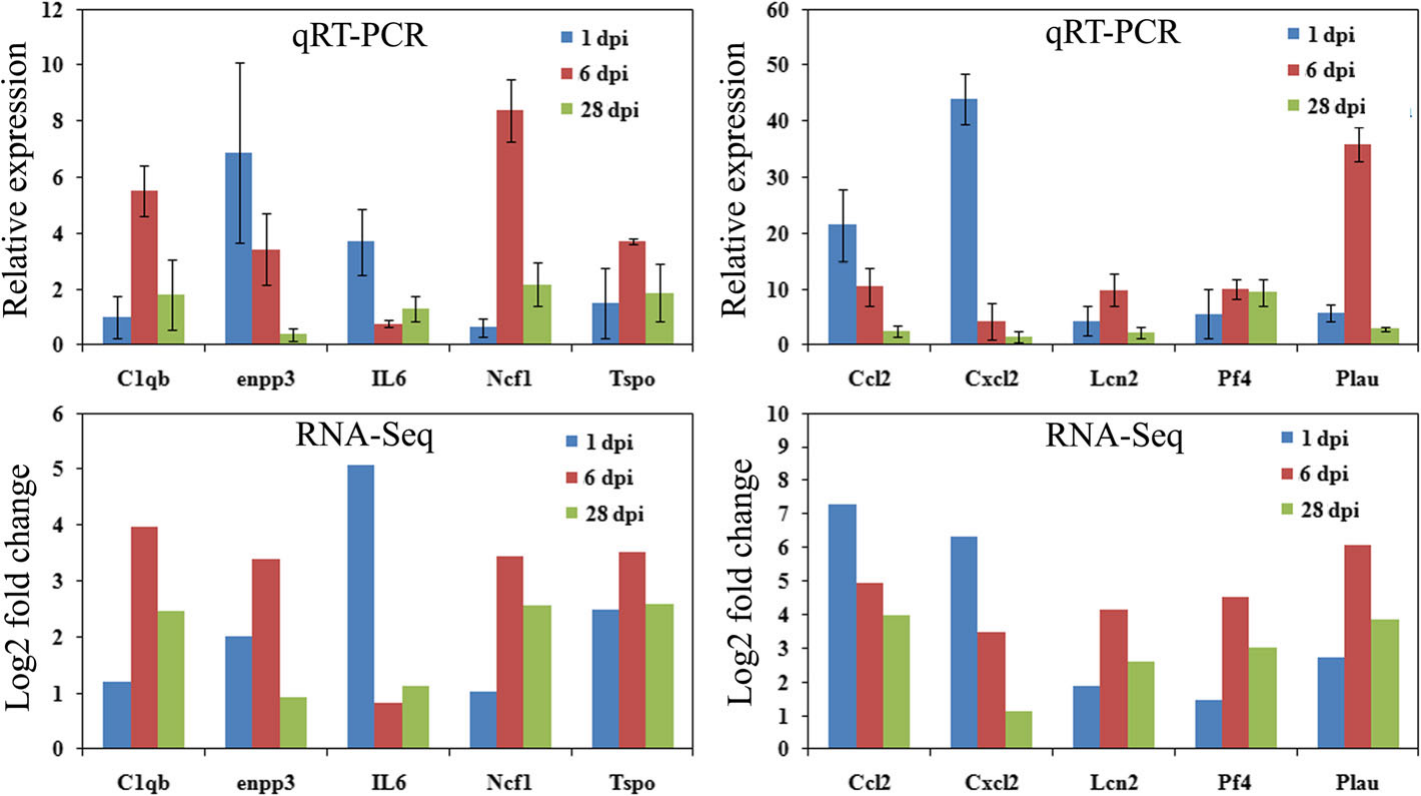
Quantitative RT-PCR validations of DEGs characterized by RNA-Seq. The relative expression level of target mRNAs was calculated using the ^ΔΔ^Ct method and expressed relative to the value in the sham group (designated as 1). Data represent the mean ± SD (*n* = 3). Log2 fold change was the ratio of average Log2 folds between samples

To verify the expression profiles obtained by RNA-Seq, ten genes were randomly selected to analyze their transcript expression levels by qRT-PCR. The selected genes included C1qb, Ccl2, Cxcl2, enpp3, IL6, Lcn2, Ncf1, Pf4, Plau, and Tspo. The results showed that these genes displayed similar expression patterns between RNA-seq and qRT-PCR (Fig. 4).

### Hierarchical cluster analysis of gene expression in spinal cords at different injury stages

**Fig. 5:**
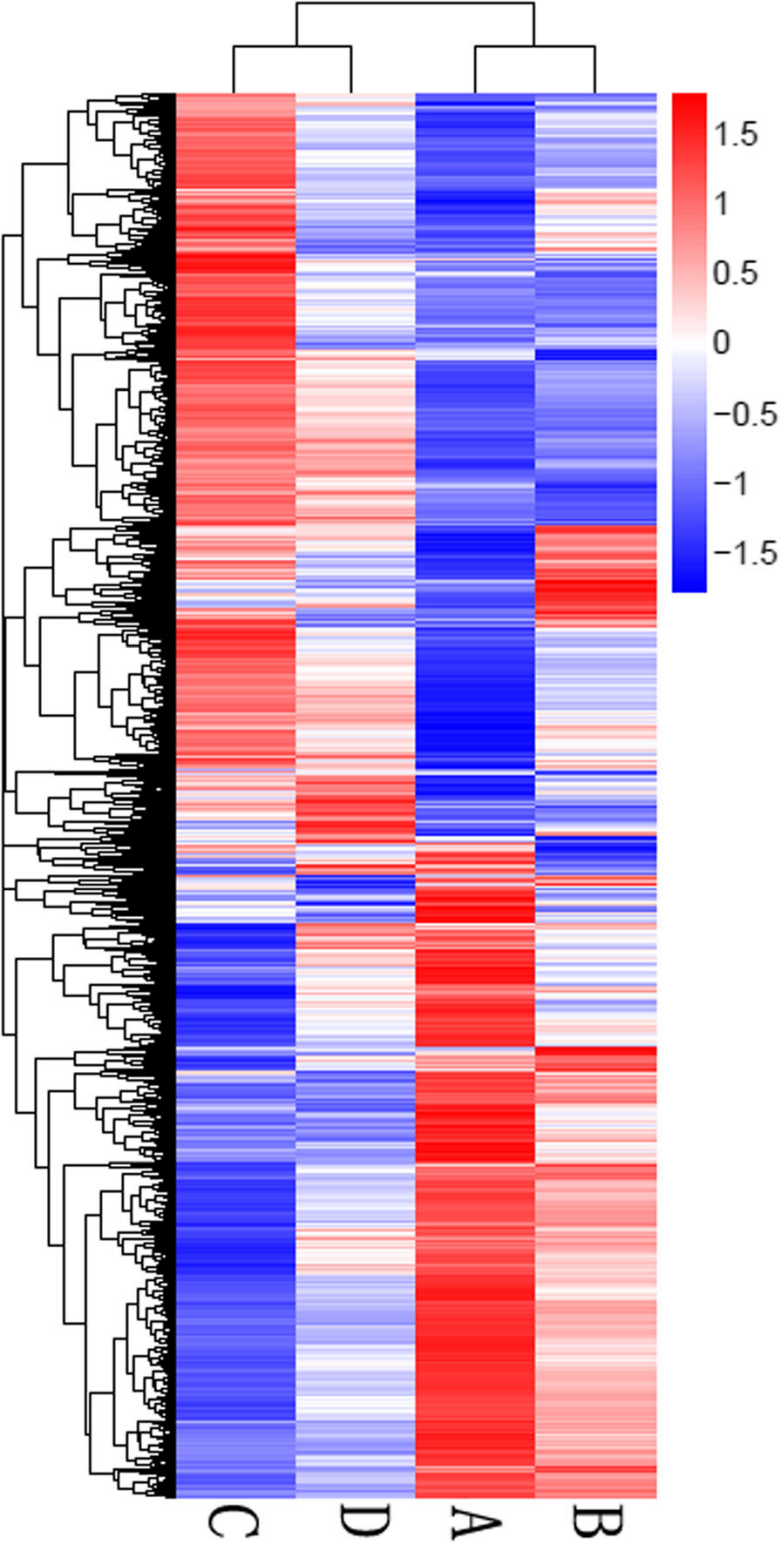
Hierarchical cluster analysis of gene expression in the spinal cords at different injured stages. Based on similarity of gene expression patterns, 7632 DEGs were classified into many expression cluster groups. The blue to red gradation represented gene expressions from down to up. **a**: sham control; **b**: 1 dpi; **c**: 6 dpi; **d**: 28 dpi

Based on the similarity of gene expression patterns, 7632 DEGs were classified into expression cluster groups by hierarchical clustering (Fig. 5). These clusters contained genes that were up- or downregulated throughout the whole course of SCI. Based on the expression patterns of these clusters, we can found that some showed marked differences between different stages of SCI, while others showed relatively minor differences. For example, most of the genes upregulated at 1 dpi (B), 6 dpi (C) and 28 dpi (D) compared to the sham group (A) were in the upper cluster, while downregulated genes were observed in the lower cluster.

### K-means clustering of DEGs

**Fig. 6:**
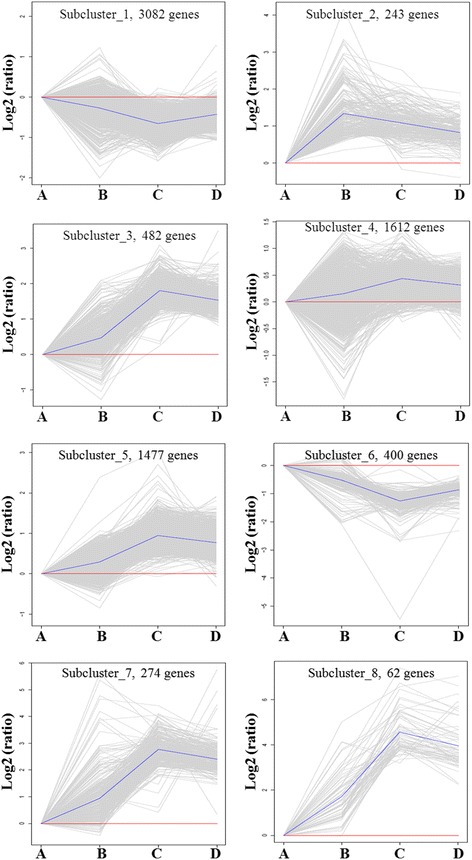
K-means clustering of DEGs. The 7632 DEGs were statistically grouped into 8 subclusters. The trends of distinct significant expression subclusters were analyzed. A: sham control; B: 1 dpi; C: 6 dpi; D: 28 dpi. (*n* = 3 in every group)

To further investigate the biological characteristics of the 7632 DEGs, K-means clustering was performed. In the analysis, DEGs were statistically grouped into eight subclusters based on their expression patterns in spinal cords at different injury stages. Figure 6 shows the trends of distinct significantly expressed subclusters of all DEGs. There were 3082 and 400 genes in subclusters 1 and 6, respectively. The gene expression levels showed a similar trend in both clusters, which were highest in sham (A), and lowest in SCI animals at 6 dpi (C). Subclusters 3, 4, 5, 7 and 8 included 482, 1612, 1477, 274 and 62 genes, respectively. The gene expression levels showed a similar trend in these five subclusters, with lowest expression in sham (A) and highest expression in SCI animals at 6 dpi (C). There were 243 genes in subcluster 2, which expressed at the lowest level in sham (A) and at the highest at 1 dpi (C) with a progressive decrease at 6 dpi (C) and 28 dpi (D). The list of gene IDs in eight subclusters are shown in Additional file 3: Table S3.

### GO enrichment of DEGs

GO enrichment analysis was conducted to characterize the DEG profiles presented above. GO enrichment results for all DEGs are provided in Additional file 4: Table S4.

In acute SCI (1 dpi), 35 significant GO terms were detected in the upregulated genes (Additional file 5: Figure S1 and Additional file 6: Table S5). No significant terms were found in the downregulated genes at 1 dpi (Additional file 7: Figure S2 and Additional file 8: Table S6).

In subacute SCI (6 dpi), 33 significant GO terms were detected in the up-regulated genes (Additional file 9: Figure S3 and Additional file 6: Table S5). Among the down-regulated genes at 6 dpi, 40 significant GO terms were detected (Additional file 10: Figure S4 and Additional file 8: Table S6).

In chronic SCI (28 dpi), 31 significant GO terms were detected in the up-regulated genes (Additional file 11: Figure S5 and Additional file 6: Table S5). Among the down-regulated genes at 28 dpi, 28 significant GO terms were detected (Additional file 12: Figure S6 and Additional file 8: Table S6).

### KEGG enrichment analysis

KEGG enrichment analysis was conducted to identify pathways that play important roles in the pathologic process of SCI. KEGG enrichment results of DEGs are shown in Additional file 13: Tables S7, Additional file 14: Table S8, and Additional file 15: Table S9.

In acute SCI (1 dpi), ribosome, TNF signaling pathway, proteoglycans in cancer, malaria, and staphylococcus aureus infection were enriched pathways in all DEGs; Of the 13 enriched pathways in upregulated DEGs, the top ten were ribosome, TNF signaling pathway, malaria, salmonella infection, leishmaniasis, HIF-1 signaling pathway, proteoglycans in cancer, leukocyte transendothelial migration, Chagas disease (American trypanosomiasis), and staphylococcus aureus infection; the 2 enriched pathways in downregulated DEGs were circadian entrainment and axon guidance (Additional file 16: Figure S7 and Additional file 13: Table S7).

In acute SCI (6 dpi), glutamatergic synapse, circadian entrainment, GABAergic synapse and retrograde endocannabinoid signaling were pathways enriched in all DEGs. The top ten pathways of the 27 enriched pathways in upregulated DEGs were ribosome, osteoclast differentiation, lysosome, NF-kappa B signaling pathway, TNF signaling pathway, Toll-like receptor signaling pathway, NOD-like receptor signaling pathway, Chagas disease (American trypanosomiasis), pertussis and salmonella infection. The top ten pathways of the 37 pathways enriched in downregulated DEGs were glutamatergic synapse, circadian entrainment, GABAergic synapse, retrograde endocannabinoid signaling, calcium signaling pathway, dopaminergic synapse, neuroactive ligand-receptor interaction, adrenergic signaling in cardiomyocytes, insulin secretion and nicotine addiction (Additional file 17: Figure S8 and Additional file 14: Table S8).

In chronic SCI (28 dpi), 39 pathways were enriched in all DEGs; the top ten were ribosome, circadian entrainment, glutamatergic synapse, GABAergic synapse, retrograde endocannabinoid signaling, salivary secretion, platelet activation, cAMP signaling pathway, nicotine addiction and lysosome; In the 21 pathways enriched in upregulated DEGs, the top ten were ribosome, lysosome, hematopoietic cell lineage, staphylococcus aureus infection, osteoclast differentiation, tuberculosis, leishmaniasis, Fc gamma R-mediated phagocytosis, phagosome and NF-kappa B signaling pathway. Of the 25 pathways enriched in downregulated DEGs, the top ten were circadian entrainment, cAMP signaling pathway, glutamatergic synapse, nicotine addiction, calcium signaling pathway, GABAergic synapse, retrograde endocannabinoid signaling, adrenergic signaling in cardiomyocytes, axon guidance and neuroactive ligand-receptor interaction (Additional file 18: Figure S9 and Additional file 15: Table S9).

## Discussion

In this study, we modeled SCI in rats using the New York University Impactor. According to the BBB scores and our previous experience [9, 15], our model produced a moderate level of impairment. To establish a systematic global analysis of gene expression patterns in the injured spinal cord, we used RNA-Seq to characterize the temporal changes after contusive SCI in rats at acute (1 dpi), subacute (6 dpi), and chronic (28 dpi) phase timepoints. The transcriptomes were systematically characterized with the goal of identifying pathways and genes critical in SCI pathology. Before data analysis, the quality of the cDNA library was examined. Our results showed that more than 77% of clean tags from RNA-Seq data mapped uniquely to the genome and that variance in the expressed transcripts as analyzed by PCA was low. These results demonstrated that our data was of sufficient quality to be used for functional analyses.

Comparing each time point to all other time points, hundreds to thousands of differentially expressed genes were obtained in the acute, subacute, and chronic phases of SCI. Hierarchical cluster analysis and K-means clustering analysis further indicated that more than 3000 genes in the two clusters decreased post-SCI and reached the lowest level at 6 dpi. There were more 4000 genes in five other clusters showing an increasing trend. Most of these genes increased post-SCI and reached the highest level at 6 dpi. There were only 243 genes in cluster 2 which expressed at the highest level at 1 dpi, followed by a decrease at 6 dpi and 28 dpi. These results suggest that our analysis may provide new information related to gene expression profiles for the study of SCI pathological mechanisms.

To further characterize the above DEG profiles, GO enrichment analysis was performed. The analysis results showed that for statistically significant differentially expressed genes, most enrichment occurred in immune response, MHC protein complex, antigen processing and presentation, translation-related genes, structural constituent of ribosome, ion gated channel activity, small GTPase mediated signal transduction, cytokine and/or chemokine activity, among others. KEGG pathway analysis showed that the top enriched pathways included ribosome, antigen processing and presentation, retrograde endocannabinoid signaling, axon guidance, dopaminergic synapses, glutamatergic synapses and GABAergic synapses, as well as TNF, HIF-1, NF-kappa B, Toll-like receptor, NOD-like receptor, cAMP, calcium, oxytocin, Rap1, B cell receptor and chemokine signaling pathway. In an early study using a mouse SCI model [16], gene profiling data at acute (2 days) and subacute phases (7 days after injury) showed that “inflammation response”, “cell death and survival”, “nervous system development” and “neurological disease” were the top enriched functional categories. “Acute Phase Response Signaling”, “LXR/RXR Activation”, “Role of Pattern Recognition Receptors in Recognition of Bacteria and Viruses” and “Atherosclerosis Signaling” were the most enriched pathways [16]. Comparing our findings to the mouse gene profiling data, we find that although animal species and data analysis methods were different, there were still many similar findings between the rat and mouse models. For example, “inflammation response” in the mouse model is similar to “immune response” in the rat model. “Role of Pattern Recognition Receptors in Recognition of Bacteria and Viruses” in mice is related to “Toll-like receptor” and “NOD-like receptor” in rats [17, 18]. These results demonstrate that there are indeed some similarities between the mechanisms of SCI in rats and mice. However, we also found many more differences in gene expression and signaling pathways between these two species. The use of high throughput RNA-Seq, coupled with different species and different laboratory results, makes it is difficult to ascertain the causes of these differences. In fact, the pathology of SCI in rats and mice is distinct, particularly with regard to cavity formation and scar/inflammatory responses, and the rat model is more similar to human SCI [19, 20]. Thus, more thorough mechanistic research using the rat model may be a better approach for understanding human SCI.

In addition to differing species, the choice detection methods may also produce important differences. There are at least four other groups that have studied gene expression during acute, subacute, and chronic SCI using methods different from RNA-Seq [21–24]. In the following sections, we will discuss the important similarities and differences in gene expression patterns in the injured spinal cord between our RNA-Seq results and data available from the other labs, as well as the advantages of RNA-Seq and the novel pathways have been discovered.

The first point we would like to make is that our results implicate many important genes previously known to be involved in SCI, such as Socs3 [25], Il17 [26], Tnf [27], Il6 [26], Il1b [28], CD44 [29], Fgf2 [30], Annexin [31], Mmp9 [32] and Bax [33]. In addition to these well-studied genes, many other genes not previously identified were found to be involved in the response to SCI. For example, in the acute phase, among the top ten significantly upregulated genes, we found that colony stimulating factor 2 receptor beta common subunit (Csf2rb), perilipin 2 (Plin2), growth arrest and DNA-damage-inducible protein GADD45 gamma (Gadd45g), strawberry notch homolog 2 (Sbno2), filamin-C (Flnc), B cell lymphoma 3 (Bcl3), glioma pathogenesis-related protein 2 (Glipr2) and tribbles pseudokinase 1 (Trib1) had not been reported in SCI.

Csf2rb is a common subunit of the GM-CSF receptor, IL-3 receptor, and IL-5 receptor [34]. GM-CSF has been shown to inhibit glial scar formation, enhance the integrity of axonal structure and produce a long-term protective effect after SCI [35]. The roles of IL-3 and IL-5 in SCI are not very clear. However, both of them and GM-CSF often co-express in TH2 cells, a subset of CD4^+^ T cells that are characterized by the production of IL-4, IL-5, IL-10 and IL-13 [36]. Therefore, in the acute phase of SCI, upregulation of their co-receptor subunit expression may be beneficial by exerting anti-inflammatory effects.

Trib1 has been shown to interact with a number of proteins, such as MEK-1, MKK4, COP1 and C/EBPalpha [37, 38]. Satoh et al. demonstrated that Trib1 plays a critical role in the differentiation of tissue-resident M2-like macrophages [39]. In our previous study, we demonstrated that macrophages can be polarized into M2 subtypes in the early stage of SCI [40]. This is consistent with the high expression of Trib1, which may play important roles in the differentiation of macrophages after SCI.

Gadd45g is involved in DNA repair, cell cycle control, cellular stress response and apoptosis, among other functions [41]. Sbno2, a novel inflammatory response factor, is predominantly expressed in astrocytes, as well as in the choroid plexus and in some microglia, endothelial cells, and neurons in the CNS [42]. Plin2, also known as adipose differentiation-related protein, is associated with adipocyte differentiation [43]. Flnc, also known as filamin-2 or actin-binding-like protein, is mainly expressed in cardiac and skeletal muscles and functions by crosslinking actin filaments and anchoring membrane proteins to the actin cytoskeleton [44]. Bcl3 is a transcriptional co-activator that is activated through interaction with p50 NF-kappaB homodimers [45]. Knockout of Bcl3 inhibited fiber atrophy and abolished NF-kappa B reporter activity [46]. Glipr2, also known as Golgi-associated plant pathogenesis-related protein 1, is a member of plant pathogenesis related proteins group 1 superfamily. It strongly associates with lipid rafts in the cytosolic leaflet of Golgi membranes [47]. However, little is known about the roles of any of these genes in SCI.

In the subacute stage, from the ten most significantly upregulated genes, we found that urokinase-type plasminogen activator (Plau), hexokinase-3 (Hk3) and triggering receptor expressed on myeloid cells 2 (Trem2) had not previously been reported in SCI. Plau is a serine protease involved in migration and proliferation of some tumor cells and degradation of the extracellular matrix [48]. Hk3, an isoform of hexokinases which catalyzes the first step of glucose metabolism, can increase ATP levels, reduce oxidant-induced reactive oxygen species, preserve mitochondrial membrane potential, increase mitochondrial biogenesis, and exert protective effects against oxidative stress [49]. Trem2, a receptor belonging to the immunoglobulin superfamily, is mainly expressed on myeloid cells where it stimulates neutrophil- and monocyte/macrophage-mediated inflammatory responses [50]. A study by Takahashi et al. reported that expression of trem2 on microglia increased their scavenging capability for apoptotic neurons without inflammation [51]. However, the functions of these genes are poorly understood in traumatic CNS injuries.

In the chronic phase, from the ten most upregulated genes, we found that tumor necrosis factor alpha-induced protein 6 (Tnfaip6), neutrophil cytosol factor 1 (Ncf1), ankyrin repeat and suppressor of cytokine signaling box protein 15 (Asb15), cupredoxins (Cp), tartrate-resistant acid phosphatase 5 (Acp5) and C-type lectin domain family 4, member A3 (Clec4a3) had not previously been reported in SCI.

Ncf1 is a cytosolic subunit of neutrophil NADPH oxidase which can be activated to produce superoxide anion. Ncf1 and NADPH oxidase 2 complex-derived reactive oxygen species are important regulators of several chronic inflammatory disorders, such as multiple sclerosis, gout, psoriasis and psoriatic arthritis, rheumatoid arthritis, and lupus [52]. Although there are no reports of increasing Ncf1 expression in SCI, it may play an important role in the chronic phase of this pathological process.

Tnfaip6, a secretory protein induced by tumor necrosis factor alpha, is involved in extracellular matrix stability and cell migration, and is important in the protease network associated with inflammation [53]. Asb15, a protein predominantly expressed in skeletal muscle, has a role in the regulation of protein turnover and muscle cell development [54]. Cps are widespread copper-binding proteins that bind a mononuclear type 1 copper redox site and are mainly involved in electron transfer reactions, photosynthesis and respiration [55]. Acp5 is involved in osteopontin/bone sialoprotein dephosphorylation, which is essential for bone resorption and osteoclast differentiation [56]. Clec4a3, a member of the C-type lectin-like domain-containing family which was originally thought to be important for initiating and shaping immune responses, has been found to be upregulated in rat spinal cord following traumatic nerve root injury [57]. However, whether these genes contribute to the pathological process of SCI remains unknown.

A second important point regarding our study is that the genes identified in SCI are involved in many signaling pathways. For example, the TNF [27, 58, 59], Toll-like receptor [60], NF-kappa B [61], NOD-like receptor [62] and synapse formation signaling pathways [63] have been reported to be involved in the pathological process of SCI [63, 64].

However, there were also some interesting new terms in SCI found by KEGG pathway analysis. For example, the ribosome term was significantly enriched in the whole pathological process. Ribosomes consist of complexes of proteins and RNAs and are complex molecular machines responsible for protein synthesis in living cells [65]. Therefore, the upregulation of ribosomal protein genes may increase production of corresponding proteins and thus boost ribosome function after SCI. The boosting function of ribosomal proteins can promote ribosome biogenesis, which may contribute to cell growth and proliferation, stress responses and the potential for repair [66]. However, the mechanisms related to ribosomes in regulating neuronal growth, stress responses and rehabilitation after SCI remain to be identified. In addition, some pathways such as retrograde endocannabinoid signaling, oxytocin signaling, Rap1 signaling and the synaptic vesicle cycle were also enriched in the subacute and chronic phases of SCI. The roles of these pathways in SCI have never been reported to our knowledge. These new findings will thus supportfuture research and therapeutic development for SCI.

## Conclusions

In summary, our study has not only characterized global changes in gene expression in injured rat spinal cords, but has also systematically identified the critical genes and signaling pathways in SCI pathology. Although the genes associated with damage and repair of spinal cord injury are still largely unknown, the RNA-Seq analysis presented in this study will expand our understanding of the complex molecular mechanisms involved and provide a foundation for future studies of tissue damage and repair after SCI.

## Additional files

Additional file 1: Table S1. Differentially expressed genes in the spinal cords at different injured stages. The downregulated genes are shown in green, the upregulated are shown in Red. (XLSX 634 kb)
Additional file 2: Table S2. The list of most relevant genes that are over-expressed or under-expressed in the different injured stages. (XLSX 44 kb)
Additional file 3: Table S3. The list of gene IDs in 8 subclusters for K-means clustering of DEGs. (XLSX 360 kb)
Additional file 4: Table S4. The significant GO terms of differentially expressed genes. (XLSX 109 kb)
Additional file 5: Figure S1. GO enrichment analysis of up-regulated genes in acute SCI (1 dpi). The 30 most enriched GO terms are shown. The asterisks (*) represent the significantly enriched (P ≤ 0.05) biological process, cellular component, and molecular function categories. (TIF 1475 kb)
Additional file 6: Table S5. GO enrichment result of the up-regulated genes. (XLSX 64 kb)
Additional file 7: Figure S2. GO enrichment analysis of down-regulated genes in acute SCI (1 dpi). The 30 most enriched GO terms are shown. No significantly enriched biological process, cellular component, and molecular function categories are detected. (TIF 1595 kb)
Additional file 8: Table S6. GO enrichment result of the down-regulated genes. (XLSX 58 kb)
Additional file 9: Figure S3. GO enrichment analysis of up-regulated genes in subacute SCI (6 dpi). The 30 most enriched GO terms are shown. The asterisks (*) represent the significantly enriched (P ≤ 0.05) biological process, cellular component, and molecular function categories. (TIF 626 kb)
Additional file 10: Figure S4. GO enrichment analysis of down-regulated genes in subacute SCI (6 dpi). The 30 most enriched GO terms are shown. The asterisks (*) represent the significantly enriched (P ≤ 0.05) biological process and molecular function categories. No significantly enriched cellular component is detected. (TIF 646 kb)
Additional file 11: Figure S5. GO enrichment analysis of up-regulated genes in chronic SCI (28 dpi). The 30 most enriched GO terms are shown. The asterisks (*) represent the significantly enriched (P ≤ 0.05) biological process, cellular component, and molecular function categories. (TIF 1644 kb)
Additional file 12: Figure S6. GO enrichment analysis of down-regulated genes in chronic SCI (28 dpi). The 30 most enriched GO terms are shown. The asterisks (*) represent the significantly enriched (P ≤ 0.05) biological process, cellular component, and molecular function categories. (TIF 1699 kb)
Additional file 13: Table S7. KEGG enrichment analysis of up DEGs in acute SCI (1 dpi). (XLSX 192 kb)
Additional file 14: Table S8. KEGG enrichment analysis of up DEGs in subacute SCI (6 dpi). (XLSX 384 kb)
Additional file 15: Table S9. KEGG enrichment analysis of up DEGs chronic SCI (28 dpi). (XLSX 267 kb)
Additional file 16: Figure S7. KEGG enrichment analysis of DEGs in acute SCI (1 dpi). The 20 most enriched KEGG pathways in 1 dpi vs sham. “Rich factor” means that the ratio of the DEGs number and the number of genes has been annotated in this pathway. The greater of the Rich factor, the greater of the degree of enrichment. (TIF 1729 kb)
Additional file 17: Figure S8. KEGG enrichment analysis of DEGs in subacute SCI (6 dpi). The 20 most enriched KEGG pathways in 6 dpi vs sham. “Rich factor” means that the ratio of the DEGs number and the number of genes has been annotated in this pathway. The greater of the Rich factor, the greater of the degree of enrichment. (TIF 1827 kb)
Additional file 18: Figure S9. KEGG enrichment analysis of DEGs in chronic SCI (28 dpi). The 20 most enriched KEGG pathways in 28 dpi vs sham. “Rich factor” means that the ratio of the DEGs number and the number of genes has been annotated in this pathway. The greater of the Rich factor, the greater of the degree of enrichment. (TIF 1748 kb)

## Abbreviations

dpi: Day postinjury
GAPDH: Glyceraldehyde-3-phosphate dehydrogenase
GO: Gene ontology
KEGG: Kyoto encyclopedia of genes and genomes
qRT-PCR: Real-time quantitative reverse transcriptase polymerase chain reaction
RNA-Seq: RNA-sequencing
SCI: Spinal cord injury
