# Supplementary figures and images for: Transcriptome profile of rat genes in injured spinal cord at different stages by RNA-sequencing
Source: BMC Genomics. 2017 Feb 15;18:173. doi: 10.1186/s12864-017-3532-x (PMC5312572; doi:10.1186/s12864-017-3532-x)

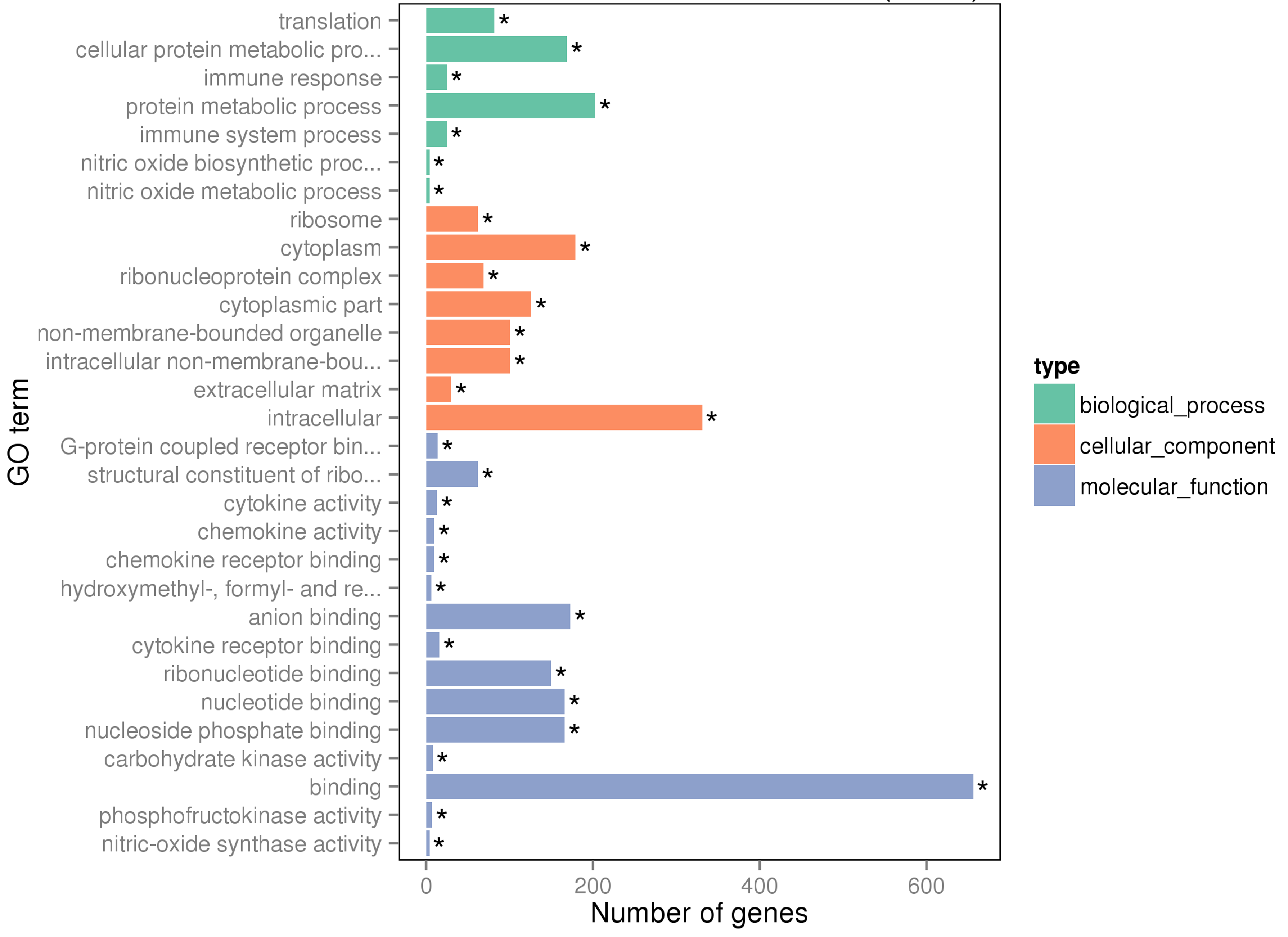

Supplement: Additional file 5: Figure S1. — GO enrichment analysis of up-regulated genes in acute SCI (1 dpi). The 30 most enriched GO terms are shown. The asterisks (*) represent the significantly enriched (P ≤ 0.05) biological process, cellular component, and molecular function categories. (TIF 1475 kb) [file 12864_2017_3532_MOESM5_ESM.tif]

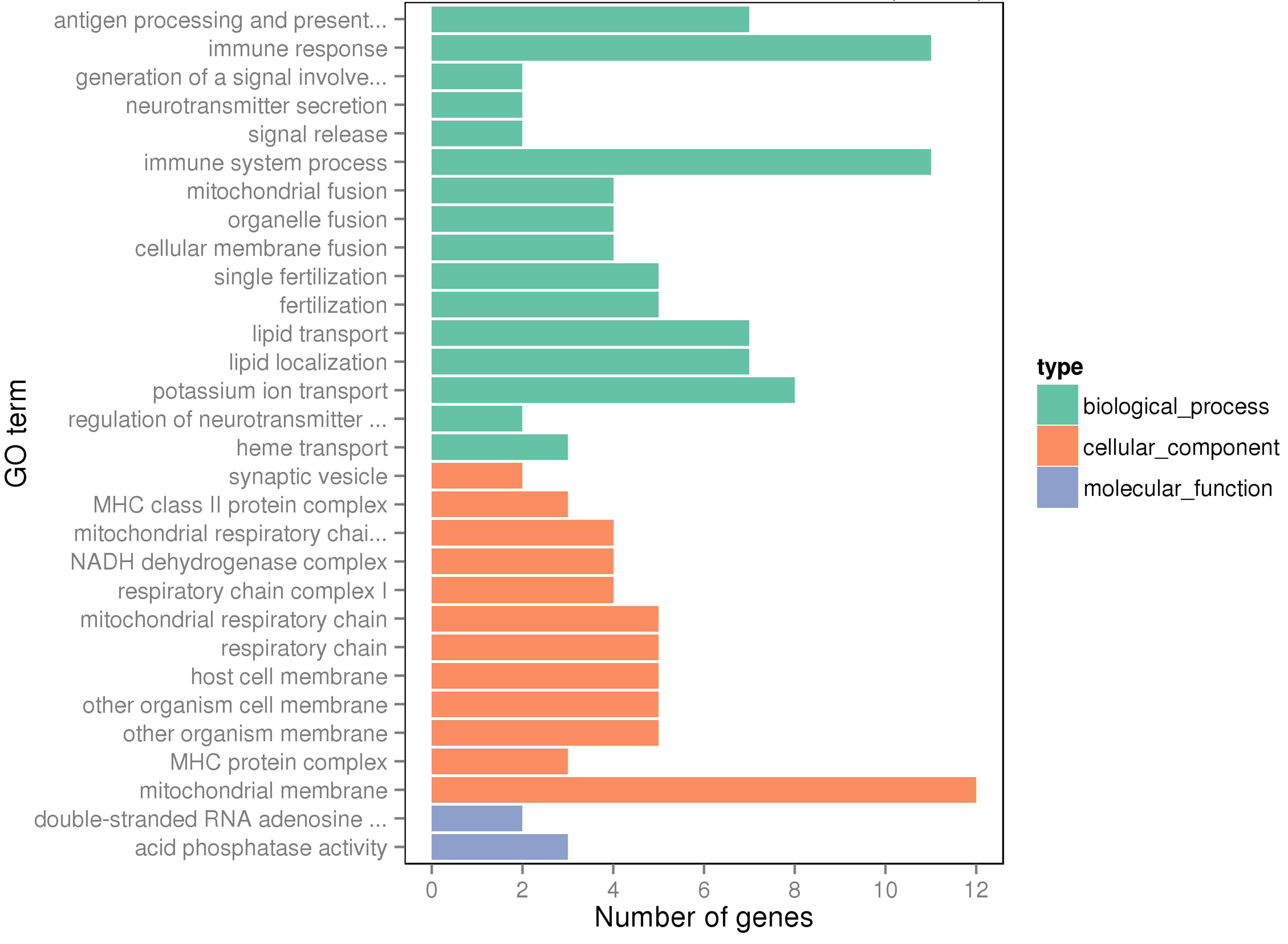

Supplement: Additional file 7: Figure S2. — GO enrichment analysis of down-regulated genes in acute SCI (1 dpi). The 30 most enriched GO terms are shown. No significantly enriched biological process, cellular component, and molecular function categories are detected. (TIF 1595 kb) [file 12864_2017_3532_MOESM7_ESM.tif]

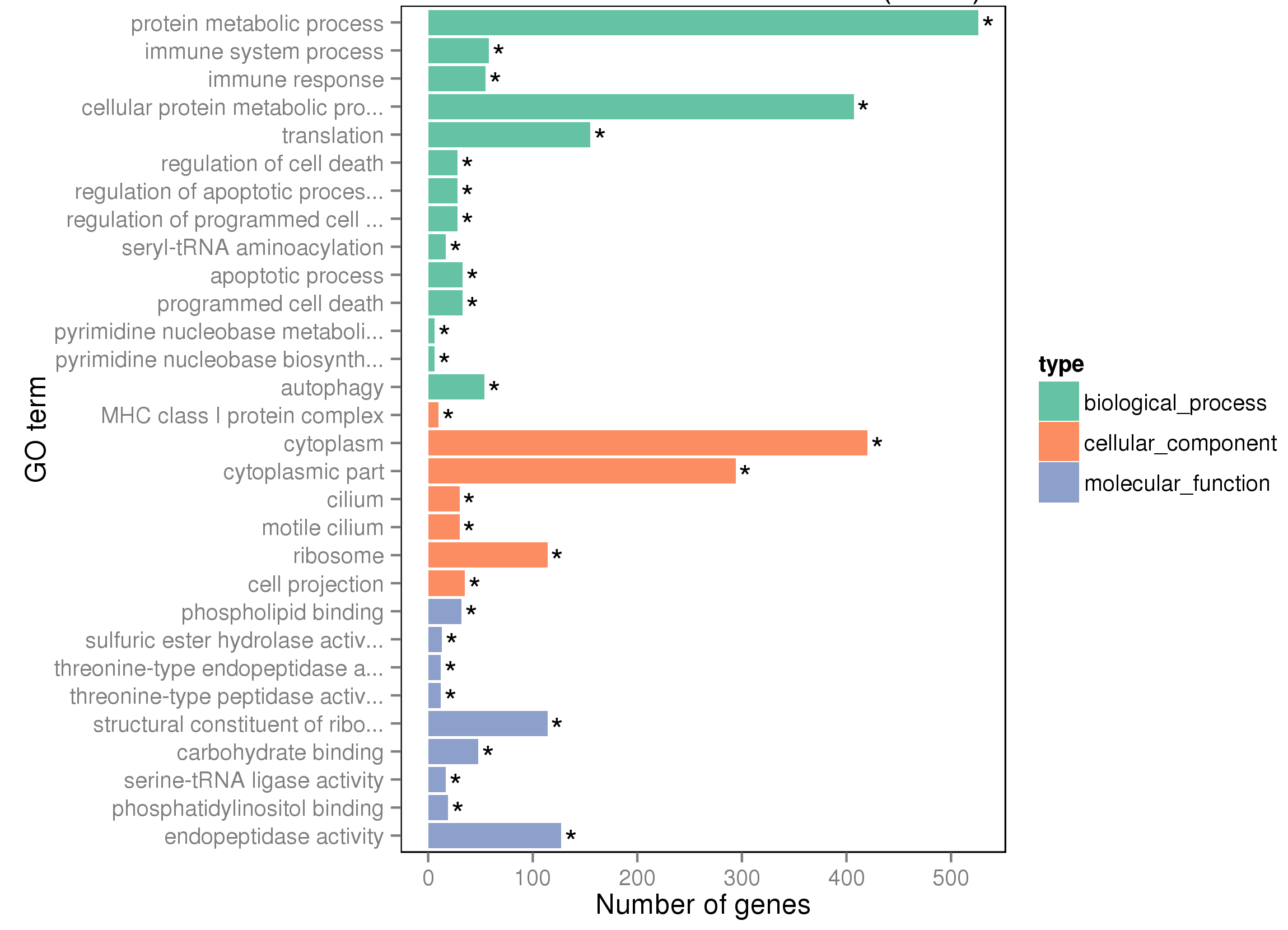

Supplement: Additional file 9: Figure S3. — GO enrichment analysis of up-regulated genes in subacute SCI (6 dpi). The 30 most enriched GO terms are shown. The asterisks (*) represent the significantly enriched (P ≤ 0.05) biological process, cellular component, and molecular function categories. (TIF 626 kb) [file 12864_2017_3532_MOESM9_ESM.tif]

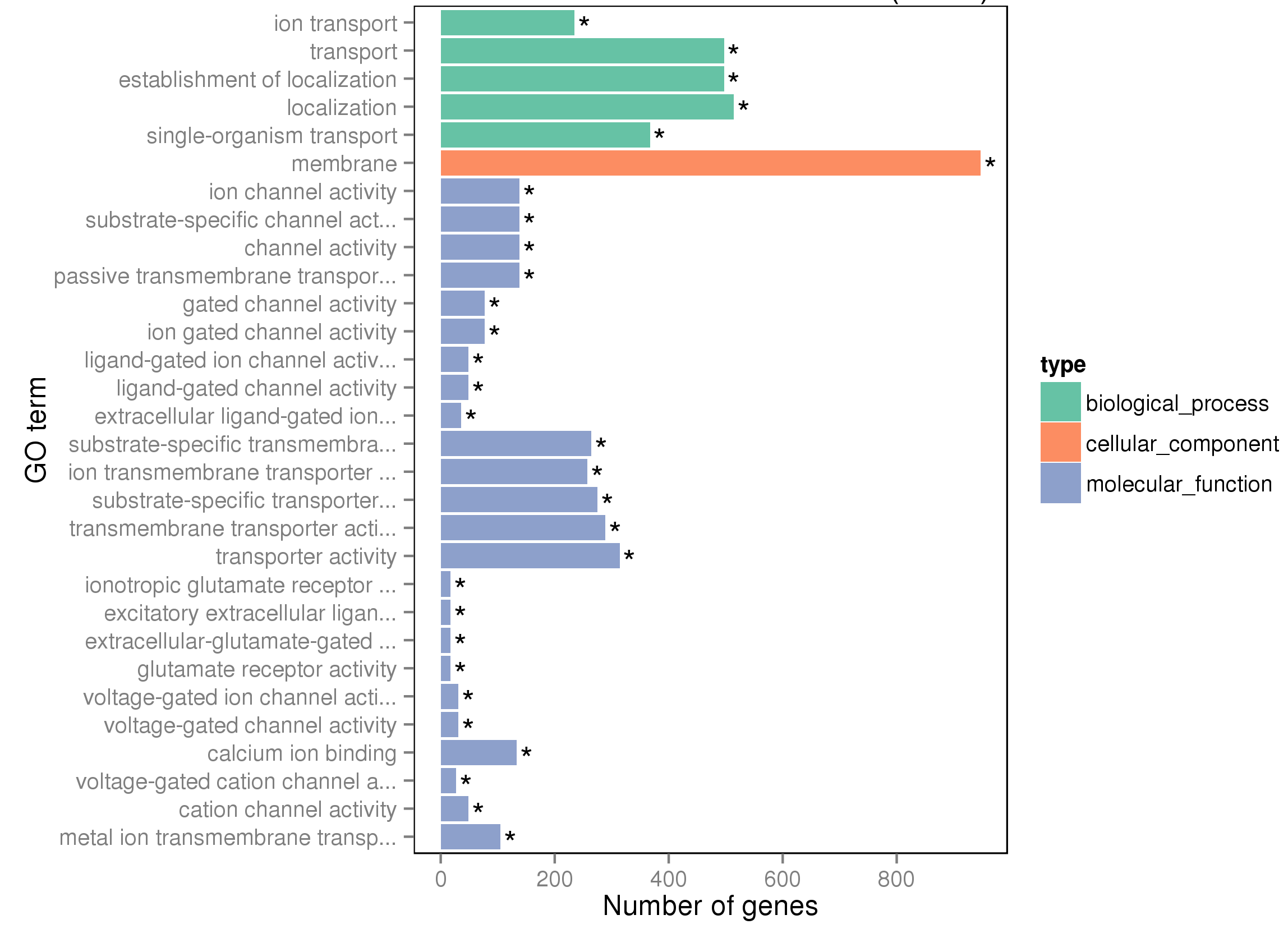

Supplement: Additional file 10: Figure S4. — GO enrichment analysis of down-regulated genes in subacute SCI (6 dpi). The 30 most enriched GO terms are shown. The asterisks (*) represent the significantly enriched (P ≤ 0.05) biological process and molecular function categories. No significantly enriched cellular component is detected. (TIF 646 kb) [file 12864_2017_3532_MOESM10_ESM.tif]

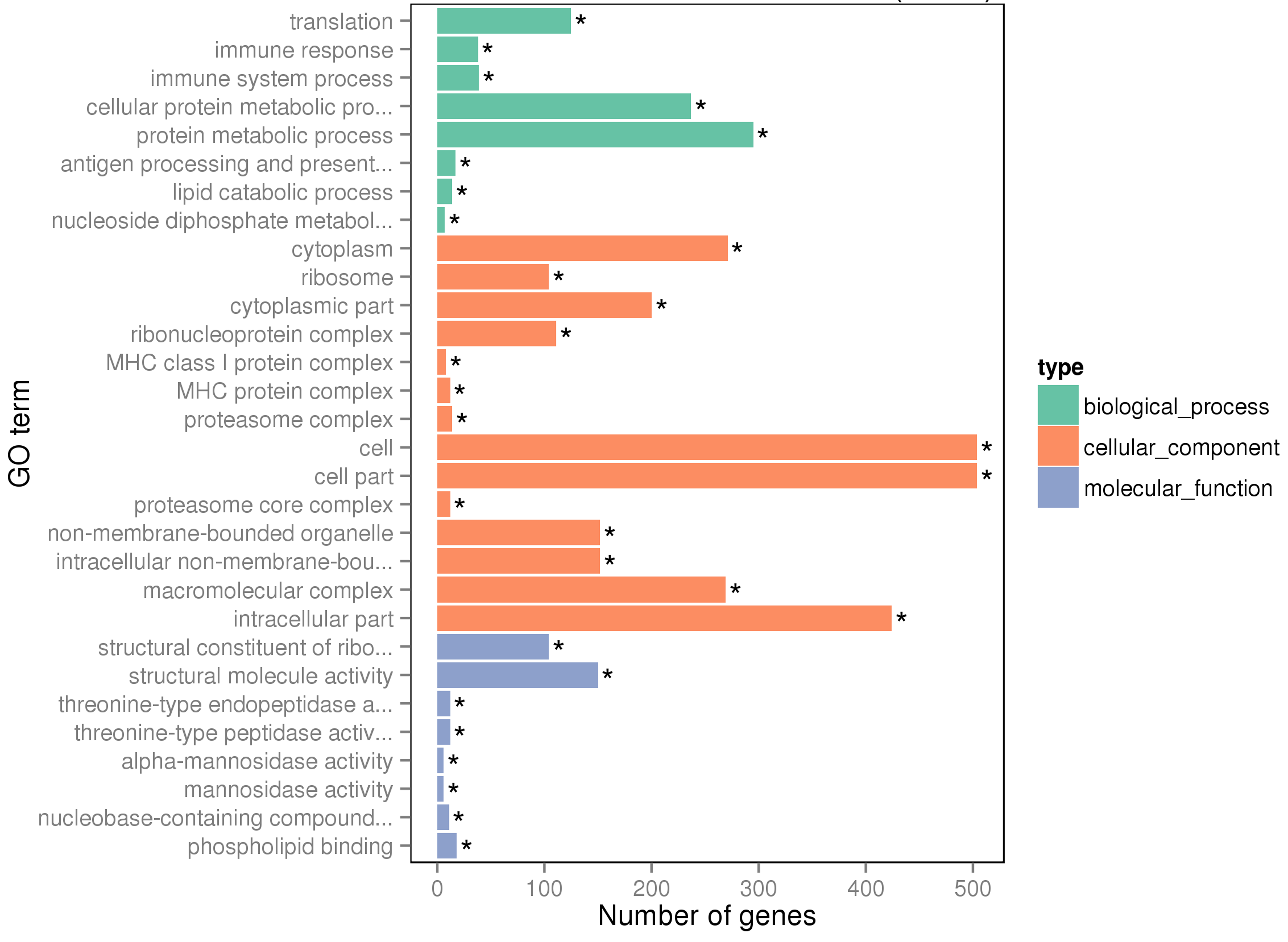

Supplement: Additional file 11: Figure S5. — GO enrichment analysis of up-regulated genes in chronic SCI (28 dpi). The 30 most enriched GO terms are shown. The asterisks (*) represent the significantly enriched (P ≤ 0.05) biological process, cellular component, and molecular function categories. (TIF 1644 kb) [file 12864_2017_3532_MOESM11_ESM.tif]

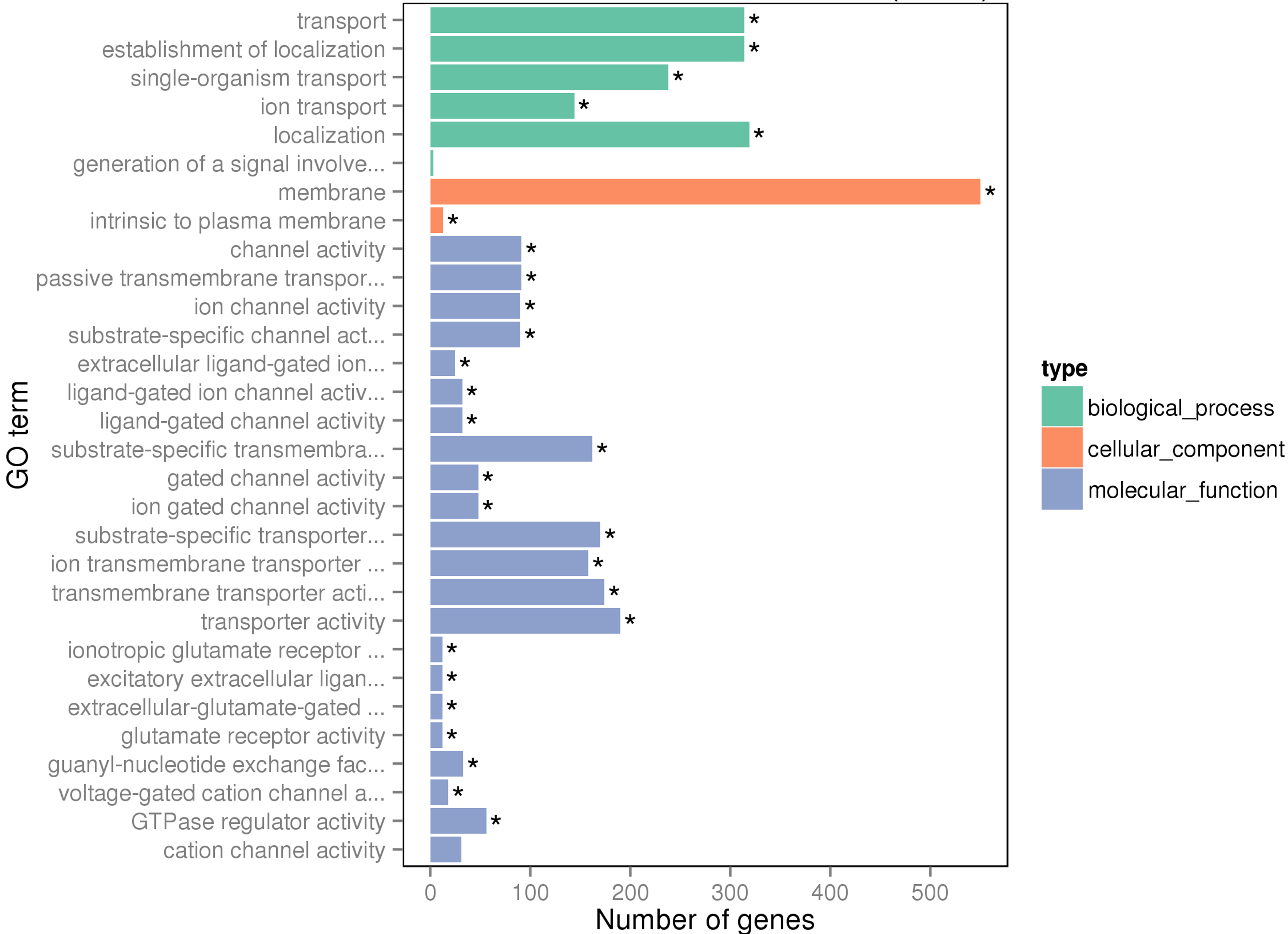

Supplement: Additional file 12: Figure S6. — GO enrichment analysis of down-regulated genes in chronic SCI (28 dpi). The 30 most enriched GO terms are shown. The asterisks (*) represent the significantly enriched (P ≤ 0.05) biological process, cellular component, and molecular function categories. (TIF 1699 kb) [file 12864_2017_3532_MOESM12_ESM.tif]

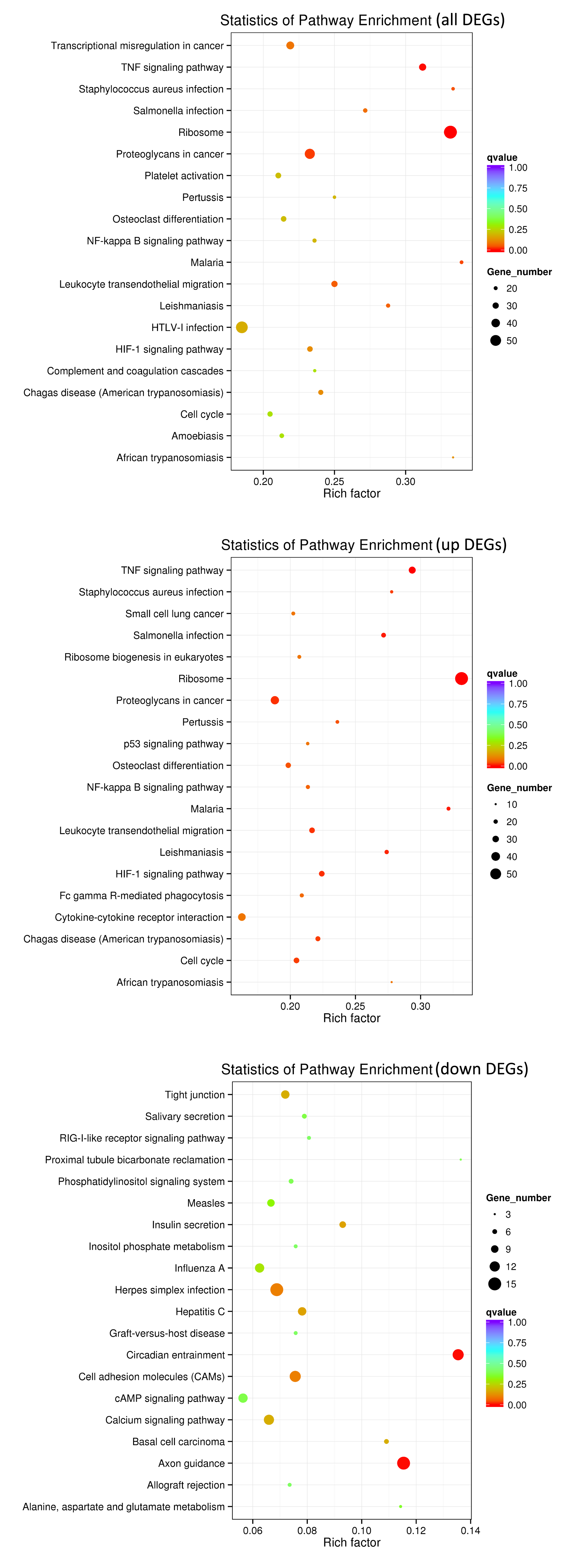

Supplement: Additional file 16: Figure S7. — KEGG enrichment analysis of DEGs in acute SCI (1 dpi). The 20 most enriched KEGG pathways in 1 dpi vs sham. “Rich factor” means that the ratio of the DEGs number and the number of genes has been annotated in this pathway. The greater of the Rich factor, the greater of the degree of enrichment. (TIF 1729 kb) [file 12864_2017_3532_MOESM16_ESM.tif]

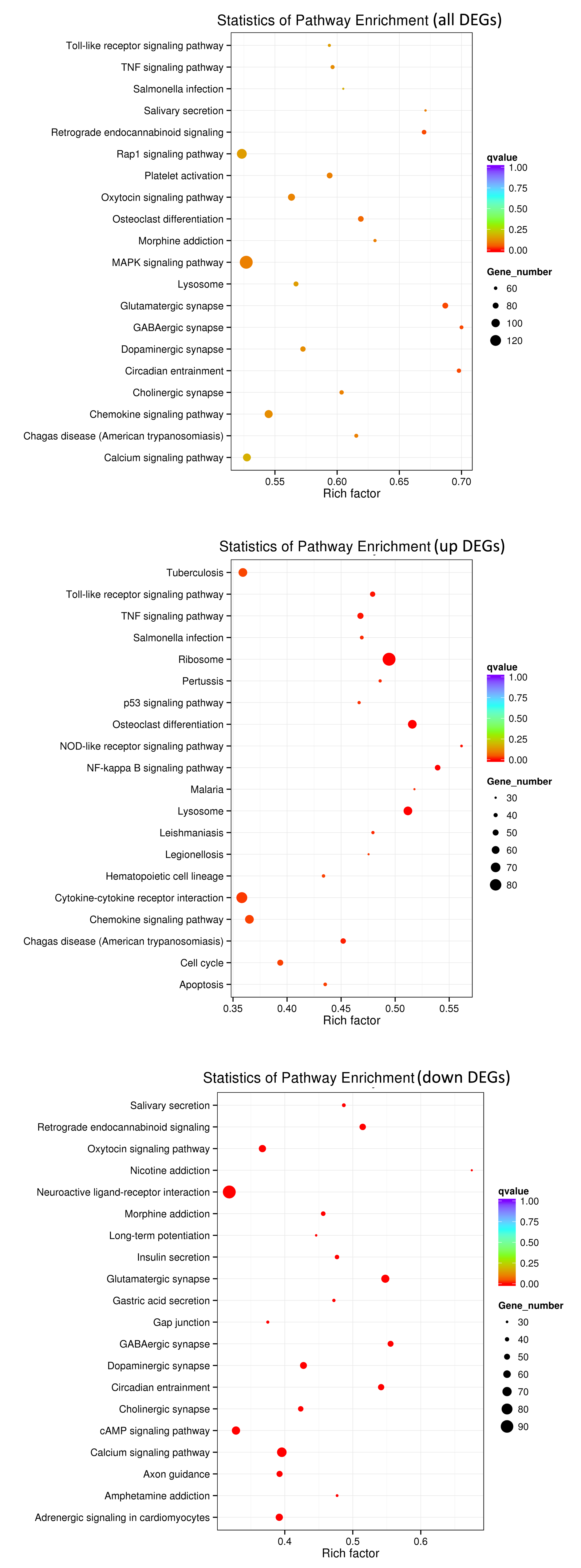

Supplement: Additional file 17: Figure S8. — KEGG enrichment analysis of DEGs in subacute SCI (6 dpi). The 20 most enriched KEGG pathways in 6 dpi vs sham. “Rich factor” means that the ratio of the DEGs number and the number of genes has been annotated in this pathway. The greater of the Rich factor, the greater of the degree of enrichment. (TIF 1827 kb) [file 12864_2017_3532_MOESM17_ESM.tif]

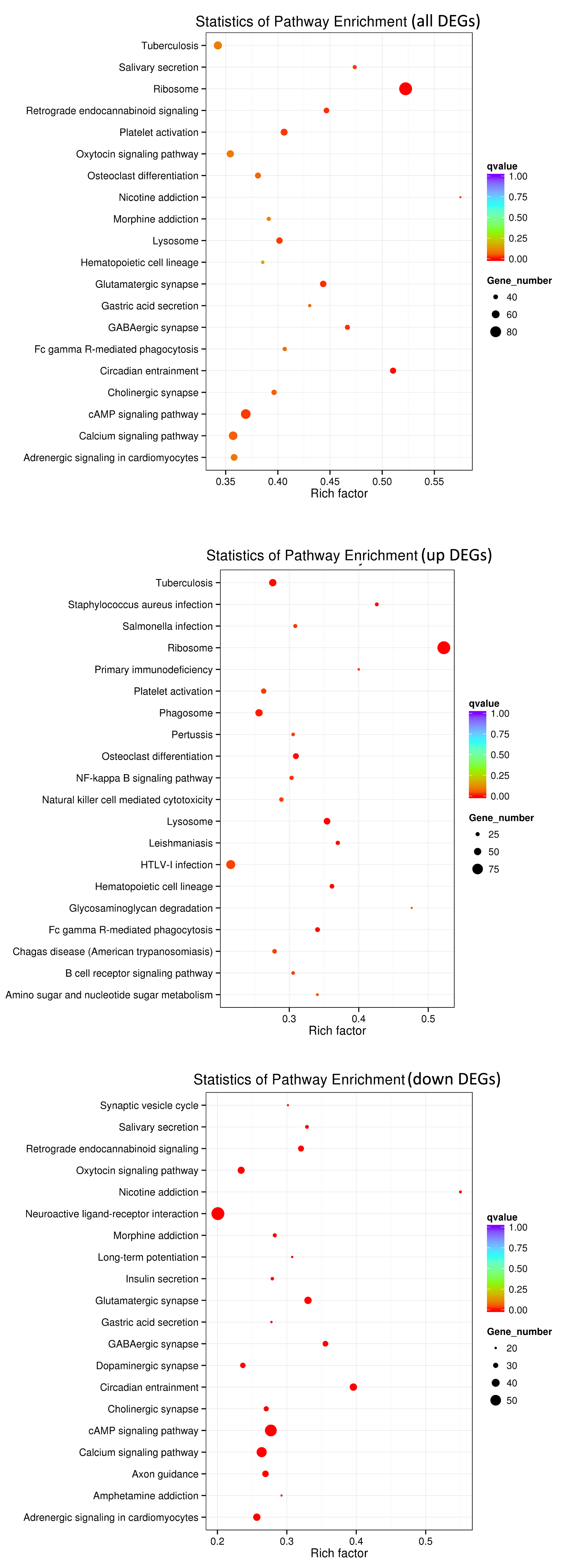

Supplement: Additional file 18: Figure S9. — KEGG enrichment analysis of DEGs in chronic SCI (28 dpi). The 20 most enriched KEGG pathways in 28 dpi vs sham. “Rich factor” means that the ratio of the DEGs number and the number of genes has been annotated in this pathway. The greater of the Rich factor, the greater of the degree of enrichment. (TIF 1748 kb) [file 12864_2017_3532_MOESM18_ESM.tif]
